# Counseling in audiology practice in Saudi Arabia: perceptions, confidence, training experiences, and barriers – a cross-sectional survey

**DOI:** 10.3389/fmed.2026.1792266

**Published:** 2026-04-21

**Authors:** Arwa Alqahtani, Basmah Almutairi, Rania Alkahtani, Reem Elbeltagy, Hadeel Alsaleh

**Affiliations:** Department of Health Communication Sciences, College of Health and Rehabilitation Sciences, Princess Nourah bint Abdulrahman University, Riyadh, Saudi Arabia

**Keywords:** counseling, audiology, confidence, training, Saudi Arabia

## Abstract

**Background:**

Counseling is a core component of audiological practice, addressing both informational and psychosocial needs of individuals with hearing loss. Despite its recognized importance, audiologists often report limited confidence in delivering personal adjustment counseling. In Saudi Arabia, evidence on counseling preparedness remains limited. This study examined audiologists' perceptions, confidence, training experiences, and perceived barriers related to counseling.

**Methods:**

A cross-sectional electronic survey was distributed to licensed audiologists across Saudi Arabia. The questionnaire assessed perceptions of counseling importance, self-reported confidence, barriers, and training experiences. Descriptive statistics, non-parametric comparisons, correlation analyses, and multiple linear regression were conducted.

**Results:**

Ninety-seven audiologists completed the survey. Counseling importance was rated high, while confidence was moderate, particularly for personal adjustment counseling skills. Barriers were generally low, though patient resistance and limited family involvement were moderately challenging. Confidence was associated with age and years of experience and was positively correlated with perceived importance and negatively correlated with perceived barriers. In the multivariable model, a perceived need for training was associated with lower confidence, while workshop participation was associated with higher confidence. Adequate training was independently associated with fewer perceived barriers.

**Conclusions:**

Audiologists in Saudi Arabia strongly value counseling but report moderate confidence, highlighting the central role of structured education and professional development in strengthening counseling competence and supporting patient-centered audiological care. However, the findings should be interpreted with caution, as the sample was predominantly composed of early- and mid-career clinicians and relied on self-reported data, which may limit generalizability to the broader national workforce.

## 1 Introduction

Counseling is an essential component of audiological practice and is explicitly included in the professional scope of both the American Speech-Language-Hearing Association (1) and the American Academy of Audiology (2). Effective counseling enables audiologists to guide patients and their families through diagnosis, management, and adjustment to hearing loss, with active family involvement further enhancing shared understanding, emotional support, adherence to audiological management, and overall wellbeing (3–5). Counseling in audiology typically involves two domains: informational counseling, which focuses on explaining test results, hearing technology, and management strategies, and personal adjustment counseling, which addresses emotional and psychosocial responses to hearing loss (4–6). Integrating both approaches is crucial for patient-centered care that meets practical and emotional needs (1, 3, 6).

Despite its importance, studies indicate that audiologists often emphasize informational counseling over personal adjustment counseling. Observational studies by Grenness et al. (7) and Ekberg et al. (8) revealed that audiologists primarily focus on providing technical information, while patients' emotional and psychosocial concerns are frequently overlooked. Similarly, Muñoz et al. (9) found that pediatric audiologists in the United States reported greater ease in performing informational counseling tasks than in addressing psychosocial challenges. This imbalance suggests that personal adjustment counseling is not consistently integrated into clinical practice.

Several factors may contribute to this gap. Time constraints are one of the most frequently cited barriers (10–13). Audiologists have also reported low confidence and insufficient skills in managing patients' emotional needs (10–14), often linked to limited formal training in counseling (10, 12, 13, 15). Variability in counseling education across audiology programs further contributes to inconsistent preparedness among graduates. For example, Whicker et al. (16) found that only three-quarters of accredited AuD programs in the United States required a dedicated counseling course, and the content and teaching methods varied considerably. Supervisors also reported feeling less confident in teaching personal adjustment counseling skills compared with informational aspects (17).

These findings suggest that while the value of counseling is widely acknowledged, audiologists face practical and educational barriers to implementing it effectively. Enhancing counseling competence requires understanding not only the training and confidence levels of audiologists but also their perceptions of counseling importance and the obstacles they encounter in practice. Although several studies have explored these aspects in Western contexts, limited evidence exists from Middle Eastern or Gulf settings, where differences in healthcare systems, patient expectations, and cultural norms may influence counseling delivery. Existing studies in the region have primarily focused on the psychosocial impact of hearing loss (18, 19), stigma related to hearing aid use (20, 21), or broader healthcare decision-making dynamics (22), rather than examining audiologists' counseling preparedness and clinical counseling practices.

In Saudi Arabia, audiological services are delivered across a mixed public–private healthcare system, with audiologists practicing in hospitals, rehabilitation centers, and private clinics. Moreover, clinical audiologists typically enter practice at the undergraduate level, placing considerable responsibility on early-career clinicians to provide comprehensive patient care, including counseling. Given the strong, culturally embedded family role in healthcare decision-making in Saudi Arabia (22), it is plausible that family involvement also shapes counseling interactions and expectations within audiology practice, particularly in pediatric contexts where parents are key partners in hearing care. Understanding how audiologists perceive their preparedness for counseling within this cultural and healthcare context is therefore essential for identifying educational gaps and supporting effective patient-centered care.

In this context, counseling competence in clinical practice may be influenced by multiple interacting factors, including clinicians' perceptions of the importance of counseling, the training they receive, their confidence in performing counseling-related tasks, and the barriers they encounter during patient interactions. Training experiences may enhance professional confidence, while perceived barriers may limit the effective implementation of counseling practices.

Therefore, this study aimed to examine audiologists' perceptions, training experiences, confidence, and perceived barriers in counseling skills within Saudi Arabia. By identifying strengths and areas needing improvement, the findings can inform counseling education and continuing professional development initiatives to enhance counseling competence and patient-centered audiologic care.

## 2 Methods

### 2.1 Study design, participants and recruitment

This study employed a cross-sectional survey design to examine audiologists' perceptions, training experiences, confidence, and barriers related to counseling practices in audiology. The target population included licensed audiologists practicing in Saudi Arabia. Audiology students and interns were excluded to ensure that responses reflected independent clinical practice.

A non-probability convenience sampling approach was used. In this method, participants are recruited based on accessibility and voluntary response rather than random selection from a defined sampling frame. Although audiologists in Saudi Arabia are licensed through the Saudi Commission for Health Specialties, the research team did not have access to a comprehensive contact list for direct recruitment. Therefore, licensed audiologists were invited to participate through professional networks and audiology-related social media platforms.

Data collection took place over a four-month period, from June to September 2025. The survey was distributed using an open-access Google Forms link. Because recruitment occurred through online dissemination and participation was voluntary, the total number of individuals who viewed the invitation could not be determined, and a response rate could not be calculated. In addition, a formal a priori sample size calculation was not performed because a complete sampling frame of licensed audiologists in Saudi Arabia was not accessible. Instead, the survey aimed to recruit as many eligible participants as possible during the data collection period.

To enhance representativeness and reduce potential sampling bias, the survey was disseminated across multiple professional networks and platforms to reach audiologists working in different regions, sectors (public and private), and levels of professional experience within Saudi Arabia.

The Google Forms platform recorded only submitted responses; therefore, only fully completed questionnaires were included in the final analysis. No partially completed surveys were retained, and no cases were excluded due to missing data. A total of 97 licensed audiologists completed the survey and were included in the final analysis.

### 2.2 Survey instrument

The survey was developed by the research team based on a review of existing literature on counseling in audiology, including previously published instruments such as Muñoz et al. (9) and was tailored to reflect counseling practices across general audiology settings within the Saudi professional context. The questionnaire was developed in English, which is the primary language of audiology education and professional practice in Saudi Arabia; therefore, translation procedures were not required. The draft instrument was reviewed by five experts in audiology and counseling to evaluate clarity, relevance, and content appropriateness within the Saudi clinical context. Based on their feedback, minor revisions were made to improve wording and item clarity. The revised survey was subsequently pilot tested with 12 practicing audiologists to assess comprehensibility and feasibility prior to final distribution. Minor refinements were implemented accordingly.

It consisted of five main sections covering demographic and professional information, perceptions of counseling aspects, counseling training, confidence in counseling skills, and perceived barriers to effective counseling (Appendix A).

Demographic questions addressed participants' age, gender, educational qualification, years of clinical experience, type of clinical setting, and region of practice. Perceptions, confidence, and barriers were assessed using 4-point Likert scales:

- Perceptions: 1 = Not important to 4 = Extremely important.
- Confidence: 1 = Not confident to 4 = Very confident.
- Barriers: 1 = Not challenging to 4 = Very challenging.

The training section explored the nature and frequency of counseling-related education received during undergraduate or postgraduate programs, clinical practice, and professional development.

### 2.3 Data analysis

The collected data were analyzed using IBM SPSS Statistics (Version 26). Prior to hypothesis testing, descriptive and preliminary analyses were conducted. Descriptive statistics, including frequencies (n) and percentages (%) were calculated for all demographic variables (gender, region, work setting, etc.) and for the categorical training variables (undergraduate/postgraduate training, career training, professional development type, etc.).

The internal consistency reliability of the three primary outcome measures (Perception, Confidence, and Barriers) was assessed using Cronbach's alpha, with coefficients of 0.64 for Perception, 0.76 for Confidence, and 0.74 for Barriers, indicating borderline internal consistency for the Perception scale and higher reliability for the Confidence and Barriers scales. Item-level descriptive statistics, including sample size (*n*), percentage (%), mean (*M*), median (Mdn), and standard deviation (SD), were calculated for all items within the three scales.

Responses were scored using a four-point Likert scale, with higher values reflecting greater perceived importance, higher confidence, or greater perceived challenge, depending on the construct being measured. Mean scores were calculated for each domain (Perceptions, Confidence, and Barriers) to provide an overall summary of responses. To interpret these scores, the scale range (1–4) was divided by the number of response categories (*k* = 4), yielding an interval width of 0.75. Based on these intervals, scores were interpreted as very low (1.00–1.75), low (1.76–2.50), moderate (2.51–3.25), and high (3.26–4.00) (23). For the Barriers domain, higher scores indicate greater perceived challenges.

Comparative analyses were conducted after assessing the assumption of normality using the Shapiro-Wilk test. Comparisons between two groups (e.g., Gender) were conducted using the Independent Samples *t*-test when assumptions of normality and homogeneity of variance were met. When the assumption of equal variances was violated, Welch's *t*-test was applied. The Mann–Whitney *U*-test was used when assumptions of normality were not satisfied. Comparisons involving three or more groups (e.g., Years of Experience) were analyzed using One-Way Analysis of Variance (ANOVA) or the Kruskal–Wallis *H*-test, as appropriate. For significant ANOVA results, Tukey's Honest Significant Difference (HSD) test was applied for post-hoc comparisons, while Bonferroni correction was used for multiple Mann–Whitney *U*-tests.

Associations among the continuous outcome variables were examined using Spearman's rank-order correlation (rho) due to non-normal data distribution. Finally, Multiple Linear Regression analyses were conducted to examine independent associations between demographic and training-related variables and the outcomes of confidence, perception, and barriers. Categorical variables (e.g., professional development type) were entered as dummy variables. Given the cross-sectional design and sample size, these analyses were performed for exploratory purposes and do not imply causal relationships. Multicollinearity among predictor variables included in the regression models was assessed using variance inflation factors (VIF) and tolerance statistics. All VIF values were below 3 and tolerance values exceeded 0.20, indicating no evidence of problematic multicollinearity among the predictors.

### 2.4 Ethical considerations

Research procedures were reviewed and approved by the Institutional Review Board (IRB) at Princess Nourah bint Abdulrahman University (Approval No. 25-0404). Participation in the electronic survey was entirely voluntary, and informed consent was obtained from all participants prior to data collection. Participants were informed of the study objectives and their right to withdraw at any time without penalty.

The survey was designed to ensure complete anonymity, as no personally identifiable information (e.g., names or contact details) was collected. All data were handled with confidentiality and were used exclusively for the academic purposes of this research.

## 3 Results

The analysis of the online questionnaire data was conducted using 97 complete responses. The demographic characteristics of the participants are presented in Table 1. The majority of the respondents were female (78.4%), predominantly aged 23–34 years (78.4%), resided in the central region (79.4%), and held a Bachelor's degree (73.2%). Clinical experience levels showed that just below half of the participants had 1–5 years of clinical experience (41%).

**Table 1:** Demographic and professional characteristics of participants (*N* = 97).

Variable	Category	*n*	%
Age	23–34 years	76	78.4
	35–45 years	17	17.5
	46–55 years	4	4.1
	56 years or older	0	0.0
Gender	Female	76	78.4
	Male	21	21.6
Highest education	Bachelor's degree	71	73.2
	Master's degree	17	17.5
	Doctoral degree	9	9.3
Years of experience	Less than 1 year	11	11.3
	1–5 years	41	42.3
	6–10 years	26	26.8
	More than 10 years	19	19.6
Primary work setting	Private clinic	37	38.1
	Public clinic	38	39.2
	Both	22	22.7
Region	Central	77	79.4
	Western	7	7.2
	Eastern	10	10.3
	Northern	0	0.0
	Southern	3	3.1

The mean scores for the three primary constructs indicated that perception of the importance of counseling was high, confidence in conducting counseling was moderate, and the perceived impact of barriers on effective counseling was low. Participants generally perceived most aspects of counseling as highly important. The only aspect rated as moderately important was “Explaining the common sociological/psychological sequelae of hearing loss to the patients” (Table 2). They rated their overall confidence in counseling skills as moderate. The lowest rated skill, indicating lower confidence among audiologists, was “Talking about the patients'/patients' family' emotions.” Additionally, participants rated themselves as moderately confident in “Assessing the presence of psychosocial challenges,” “Assessing the need for psychological referral,” and “Assessing the patient's understanding of technical information (HAs/CIs)” (Table 3). No aspect of barriers was rated as highly challenging. However, two factors were rated as moderately challenging and affecting effective counseling: “The patient's resistance to counseling” and “Limited family involvement” (Table 4).

**Table 2:** Audiologists' ratings of their perception of the importance of different counseling aspects.

Item	Not Important	Slightly important	Moderately important	Extremely important	*M*	Mdn	SD	Rank
	*N* (%)							
Explaining the diagnosis of hearing loss and related symptoms to the patient?	0 (0)	1 (1)	3 (3.1)	93 (95.9)	3.95	4	0.27	High
Explaining the expectations (e.g., outcomes, process, realistic goals) to the patient?	1 (1)	2 (2.1)	11 (11.3)	83 (85.6)	3.81	4	0.51	High
Discussing psychological referral for those who experience significant mental health challenges?	1 (1)	10 (10.3)	30 (30.9)	56 (57.7)	3.45	4	0.72	High
Explaining the technical information (related to Hearing aids/implantable devices) to the patients?	2 (2.1)	8 (8.2)	34 (35.1)	53 (54.6)	3.42	4	0.73	High
Discussing patients' and their families' emotions when receiving a diagnosis (e.g., sadness, anger, and confusion)?	4 (4.1)	7 (7.2)	37 (38.1)	49 (50.5)	3.35	3	0.79	High
Explaining the common sociological/psychological sequelae of hearing loss (e.g., social isolation, depression, stress) to the patient?	6 (6.2)	9 (9.3)	37 (38.1)	45 (46.4)	3.25	4	0.87	Moderate
Mean= 3.54 (high)								

**Table 3:** Audiologists' ratings of their confidence in conducting counseling.

Item	Not confident	Somewhat confident	Confident	Very confident	Mean	Median	SD	Rank
	*N* (%)							
Talking about the patients\ patients' family' emotions (e.g., sadness, anger)	10 (10.3)	30 (30.9)	31 (32)	26 (26.8)	2.75	3	0.69	Low
Assessing the patient's understanding of technical information (HAs\CIs)	0 (0)	15 (15.5)	45 (46.1)	37 (38.1)	3.22	3	0.69	Moderate
Assessing the presence of psychosocial challenges	9 (9.3)	36 (37.1)	33 (34)	19 (19.6)	2.63	3	0.90	Moderate
Explaining the diagnosis of hearing loss and related symptoms to the patients	0 (0)	3 (3.1)	17 (17.5)	77 (79.4)	3.76	4	0.49	High
Explaining the expectations (e.g., outcomes, process, realistic goals) to the patient	3 (3.1)	7 (7.2)	31 (32)	56 (57.7)	3.44	4	0.76	High
Assessing the need for psychological referral	12 (12.4)	40 (41.2)	22 (22.7)	23 (23.7)	2.57	2	0.98	Moderate
Mean= 3.07 (Moderate)								

**Table 4:** Audiologists' ratings of the barriers faced in counseling practice.

Item	Not challenging	Somewhat challenging	Challenging	Very challenging	Mean	Median	SD	Rank
	*N* (%)							
Not having sufficient time to address the patient's emotional needs	8 (8.2)	46 (47.4)	31 (32)	12 (12.4)	2.48	2	0.81	Low
Not having the confidence to perform proper counseling	47 (48.5)	38 (39.2)	10 (10.3)	2 (2.1)	1.65	2	0.74	Very low
Not receiving enough counseling training	28 (28.9)	38 (39.2)	18 (18.6)	13 (13.4)	2.16	2	0.99	Low
Limited educational resources for the patients	12 (12.4)	43 (44.3)	29 (29.9)	13 (13.4)	2.44	2	0.87	Low
The patient's resistance to counseling (e.g., Denial, or low motivation)	6 (6.2)	30 (30.9)	43 (44.3)	18 (18.6)	2.75	3	0.82	Moderate
Difficulty managing patient and family expectations	15 (15.5)	46 (47.4)	30 (30.9)	6 (6.2)	2.27	2	0.80	Low
Limited family involvement (e.g., Lack of family\partner support)	16 (16.9)	31 (32)	24 (24.7)	26 (26.8)	2.61	3	1.05	Moderate
Mean= 2.34 (Low)								

The training experiences of the participants are summarized in Table 5. Just below half of the participants (44.3%) reported being taught counseling skills as part of 1–2 audiology courses during their undergraduate or postgraduate studies. Only 10.3% reported having a dedicated course for counseling. During their careers, approximately 60% of participants reported having no training or rarely trained. Regarding training adequacy, 37% found the training adequate, while 40% found it inadequate. Interestingly, only 7.2% reported being fully competent to conduct counseling, whereas 46.4% reported they would benefit from additional training, and 16.5% reported needing extensive training.

**Table 5:** Counseling training experiences of audiologists.

Variable	Category	*n*	%
Undergraduate/postgraduate training	No training received	12	12.4
	Included in 1–2 audiology courses	43	44.3
	Included in several audiology courses	32	33.0
	Dedicated counseling course	10	10.3
Training during career	No training	28	28.9
	Rarely	30	30.9
	Occasionally	23	23.7
	Frequently/Very frequently	16	16.5
Professional development^*^	Workshops	21	21.6
	Online courses	25	25.8
	Peer mentorship/shadowing	46	47.4
	None	29	29.9
Adequacy of training	Very inadequate	11	11.3
	Inadequate	18	18.6
	Neutral	31	31.9
	Adequate	30	31.0
	Very adequate	7	7.2
Need for further training	Fully competent	7	7.2
	Occasional refreshers	29	29.9
	Would benefit from additional training	45	46.4
	Need extensive training	16	16.5

^*^Participants are allowed to select more than one option.

### 3.1 Comparative analysis of demographic groups

#### 3.1.1 Perception scores

No statistically significant difference was found in Perception scores across the groups of all demographic variables (Table 6). This suggests that demographics do not appear to be a significant factor in the perceived importance of counseling.

**Table 6:** Comparitive analysis of the perception scores across the groups of all demographic variables.

Variable	*n*	Mean rank	Mdn	Statistic	*p*
Age					
23–34	76	47.93	3.6	^*^X^2^(2) = 0.605	0.739
35–45	17	51.97	3.7		
46–55	4	56.63	3.7		
Education					
Bachelor	71	46.56	3.5	^*^X^2^(2) = 5.1	0.07
Masters	17	48.65	3.6		
Doctoral	9	68.89	3.8		
Years of experience					
Less than 1 year	11	39.91	3.3	^*^X^2^(3) = 2.179	0.536
2–5 years	41	47.26	3.6		
6–10 years	26	53	3.7		
More than 10 years	19	52.55	3.7		
Work settings					
Public	38	46.09	3.55	^*^X^2^(2) = 3.128	0.209
Private	37	46.5	3.5		
Both	22	58.23	3.8		
Region					
Central	77	50.77	3.7	^*^X^2^(3) = 7.189	0.066
Western	7	22.86	3.3		
Eastern	10	49.65	3.55		
Northern	3	62.5	3.8		
Southern	-	-	-		
Gender					
Male	21	45.69	3.5	^**^U = 728.50, z = −0.615	0.538
Female	76	49.91	3.7		

^*^Kruskal-Wallis *H-*test was used to compare perception scores across the different groups of age, educational level, years of experience, work settings and region of residency. ^**^The Mann-Whitney *U*-test was used to compare perception scores across the two groups of gender.

#### 3.1.2 Confidence scores

Comparison of Confidence scores across demographic groups indicated that age [X^2^(2) = 8.711, *p* = 0.013] and years of experience [X^2^(3) = 10.805, *p* = 0.013] were the only statistically significant factors (Table 7).

- Age: *Post-hoc* comparisons (Mann-Whitney *U*-test with Bonferroni correction, α = 0.017) revealed a statistically significant difference in Confidence scores only between Age Group (23–34) (median = 3.0) and Age Group (35–45) (median = 3.5) (*U* = 352, *z* = −2.945, *p* = 0.003). The Age Group (35–45) was the significantly more confident group. Comparisons involving Age Group (46–55) were not statistically significant (*p* > 0.017), though this must be interpreted with caution due to the severe limitation of the small sample size (*n* = 4) in that group.
- Years of Experience: *Post-hoc* pairwise comparisons (Mann-Whitney *U*-test with Bonferroni correction, α = 0.0083) identified only one statistically significant pair: the 2–5 years group (median = 3.0) was significantly less confident than the More than 10 years group (median = 3.5) (*p* = 0.004). This finding suggests that the only reliable difference in confidence exists between the earliest and most veteran career stages. Confidence levels of the less than one year and 6–10 years practitioners were statistically indistinguishable from their adjacent groups.

**Table 7:** Comparitive analysis of the confidence scores across the groups of all demographic variables.

Variable	*n*	Mean rank	Mdn	Statistic	*p*
Age					
23–34	76	45.22	3	^*^X^2^(2) = 8.711	0.013 ^*^
35–45	17	67.15	3.5		
46–55	4	43.63	2.8		
Education					
Bachelor	71	46.75	3	^*^X^2^(2) = 3.612	0.164
Masters	17	49.68	3		
Doctoral	9	65.5	3.5		
Years of experience					
Less than 1 year	11	44.68	3	^*^X^2^(3) = 10.805	0.013 ^*^
2–5 years	41	39.54	3		
6–10 years	26	56.33	3.15		
More than 10 years	19	61.89	3.3		
Work settings					
Public	38	49.88	3	^*^X^2^(2) = 2.279	0.320
Private	37	44.26	3		
Both	22	55.45	3		
Region					
Central	77	49.31	3	^*^X^2^(3) = 1.491	0.684
Western	7	38.29	3		
Eastern	10	51.05	3.05		
Northern	3	59.17	3.6		
Southern	-	-	-		
Gender					
Male	21	59.19	3.3	^**^U = 584, z = −1.889	0.059
Female	76	46.18	3		

^*^Kruskal-Wallis *H*-test was used to compare Confidence scores across the different groups of age, educational level, years of experience, work settings and region of residency. ^**^The Mann-Whitney *U*-test was used to compare Confidence scores across the two groups of Gender. Bold values indicate statistically significant results (*p* < 0.05).

#### 3.1.3 Barriers scores

No statistically significant difference was found in Barriers scores across the groups of all demographic variables (Table 8). This indicates that demographics do not appear to be a significant factor in the perceived barriers faced.

**Table 8:** Comparitive analysis of the barriers scores across the groups of all demographic variables.

Variable	*n*	M (SD)	Mdn	Statistic	*p*
Age					
23–34	76	2.37 (0.52)	2.4	^*^ANOVA F = 1.393	0.253
35–45	17	2.141 (0.67)	2.0		
46–55	4	2.5 (0.33)	2.5		
Education					
Bachelor	71	2.3 (0.53)	2.4	^*^ANOVA F = 1.149	0.321
Masters	17	2.4 (0.58)	2.4		
Doctoral	9	2.07 (0.65)	2.1		
Years of experience					
Less than 1 year	11	2.66 (0.54)	2.6	^*^ANOVA F = 1.727	0.167
2–5 years	41	2.34 (0.46)	2.3		
6–10 years	26	2.25 (0.58)	2.35		
More than 10 years	19	2.23 (0.65)	2.1		
Work settings					
Public	38	2.38 (0.57)	2.4	^*^ANOVA F = 0.289	0.750
Private	37	2.3 (0.44)	2.3		
Both	22	2.29 (0.68)	2.4		
Region					
Central	77	2.29 (0.51)	2.3	^*^ANOVA F = 1.181	0.321
Western	7	2.5 (0.58)	2.5		
Eastern	10	2.4 (0.51)	2.25		
Northern	3	2.8 (1.3)	3.0		
Southern	–	–	–		
Gender					
Male	21	2.25 (0.78)	2.1	^**^t_(95)_ = −0.592	0.559
Female	76	2.35 (0.47)	2.4		

^*^One way ANOVA was used to compare Barriers scores across the different groups of age, educational level, years of experience, work settings and region of residency. ^**^The Independent Sample *t*-test was used to compare Barriers scores across the two groups of gender.

Effect size estimates indicated negligible to small effects across most demographic comparisons (ε^2^ range = 0.00–0.08; η^2^ range = 0.01–0.05; *r* = 0.06–0.19), with moderate effects observed only for age and years of experience in relation to confidence scores (ε^2^ = 0.07–0.08). These results suggest that demographic differences generally had limited practical impact on counseling perceptions, confidence, or perceived barriers among participants.

A Spearman's Rho correlation was conducted to examine the relationships among the three continuous variables. A weak to moderate, positive, and statistically significant correlation was found between Perception and Confidence (rho = 0.345, *p* = 0.001). This indicates that a higher perceived importance of counseling is reliably associated with greater professional confidence. Additionally, a weak, negative, and statistically significant correlation was found between Confidence and Barriers (rho = −0.244, *p* = 0.016). This suggests that as confidence increases, the perceived level of barriers tends to decrease. The relationship between Perception and Barriers was negligible and not statistically significant (rho = −0.009, *p* = 0.929).

Multiple Linear Regression was employed to examine independent associations between the study variables and the three main outcomes.

### 3.2 Associations with confidence

The overall regression model examining Confidence was statistically significant [*F*_(10, 86)_ = 4.29, *p* = 0.001] and explained 33.3% of the variance in Confidence (Adjusted R^2^ = 0.333). Three variables showed statistically significant independent associations with Confidence (Table 9).

- A higher self-assessed need for training was independently associated with lower Confidence (β= −0.318, *p* = 0.002).
- Participation in workshops for professional development was independently associated with higher Confidence (β = 0.328, *p* = 0.005).
- Perceived importance of counseling was positively associated with Confidence (β = 0.320, *p* = 0.001).

**Table 9:** Multiple linear regression analysis of factors independently associated with professional confidence scores.

Independent variable	B	SE B	β	*t*	*p*
Perception_mean	0.446	0.127	0.320	3.502	0.001 ^*^
Barriers_mean	−0.192	0.099	−0.189	−1.931	0.057
Training during studies	−0.022	0.056	−0.038	−0.392	0.697
Training during career	0.046	0.060	0.090	0.770	0.443
Adequacy of training	−0.062	0.060	−0.104	−1.031	0.306
Need for training	−0.253	0.077	−0.318	−3.280	0.002 ^*^
Training through workshops	0.382	0.132	0.328	2.892	0.005 ^*^
Training through online courses	−0.124	0.125	−0.108	−0.988	0.326
Training through peer/mentor shadowing	0.22	0.129	0.020	0.174	0.862
No training	0.081	0.206	0.055	0.394	0.695

R^2^ = 0.333, *F*_(10, 86)_ = 4.29, *p* < 0.001. Bold values indicate statistically significant results (*p* < 0.05).

### 3.3 Associations with perception

The overall regression model examining Perception was statistically significant [*F*_(10, 86)_ = 1.958, *p* = 0.048] and explained 18.5% of the variance in Perception (Adjusted R^2^ = 0.185). Confidence was the only variable that showed a statistically significant association with Perception (β = 0.390, *p* = 0.001) (Table 10). This finding suggests that higher professional confidence was associated with a stronger perceived importance of counseling. Training variables and Barriers were not significantly associated with Perception in the multivariable model.

**Table 10:** Multiple linear regression analysis of factors independently associated with professional perception scores.

Independent variable	B	SE B	β	*t*	*p*
Confidence_mean	0.280	0.080	0.390	3.502	0.001 ^*^
Barriers_mean	0.073	0.080	0.100	0.907	0.367
Training during studies	0.045	0.044	0.108	1.014	0.313
Training during career	0.041	0.047	0.110	0.857	0.394
Adequacy of training	0.039	0.048	0.091	0.812	0.419
Need for training	0.017	0.065	0.031	0.269	0.788
Training through workshops	−0.046	0.110	−0.055	−0.0420	0.676
Training through online courses	0.060	0.100	0.074	0.606	0.546
Training through peer/mentor shadowing	0.028	0.102	0.035	0.274	0.785
No training	0.121	0.163	0.115	0.740	0.461

R^2^ = 0.185, *F*_(10, 86)_ = 1.958, *p* = 0.048. Bold values indicate statistically significant results (*p* < 0.05).

### 3.4 Associations with barriers

The overall regression model examining Barriers was statistically significant [*F*_(10, 86)_ = 2.496, *p* = 0.011] and explained for 22.5% of the variance in Barriers (Adjusted R^2^ = 0.225). Two training-related variables showed statistically significant independent associations with Barriers (Table 11):

- Greater perceived adequacy of training was independently associated with lower Barriers scores (β = −0.302, *p* = 0.005). Given the scale of the outcome (1 = No Barriers), this indicates that higher perceived adequacy of training was associated with fewer perceived barriers.
- Reporting no professional development was independently associated with higher Barriers scores (β = 0.325, *p* = 0.030), demonstrating that participants who reported receiving no professional development reported higher perceived Barriers scores compared to those who received some form of training.

**Table 11:** Multiple linear regression analysis of factors independently associated with barriers scores.

Independent variable	B	SE B	β	*t*	*p*
Perception_mean	0.130	0.144	0.095	0.907	0.367
Confidence_mean	−0.216	0.112	−0.220	−1.931	0.057
Training during studies	0.036	0.059	0.064	0.608	0.545
Training during career	0.095	0.063	0.188	1.514	0.134
Adequacy of training	−0.176	0.061	−0.302	−2.878	0.005 ^*^
Need for training	0.034	0.087	0.044	0.394	0.695
Training through workshops	0.083	0.147	0.073	0.568	0.571
Training through online courses	−0.050	0.134	−0.045	−0.375	0.708
Training through peer/mentor shadowing	−0.008	0.136	−0.007	−0.056	0.955
No training	0.470	0.213	0.325	2.205	0.030 ^*^

R^2^ = 0.225, *F*_(10, 86)_ = 2.496, *p* = 0.011. Bold values indicate statistically significant results (*p* < 0.05).

These findings suggest that both the perceived quality and the presence of training were independently associated with perceived professional obstacles, although causal inferences cannot be drawn from the cross-sectional design.

## 4 Discussion

The findings of this study provide important insight into how audiologists in Saudi Arabia perceive, implement, and are supported in counseling practices within clinical care. Although participants demonstrated a strong appreciation for the importance of counseling, as reflected in the consistently high perception ratings (Table 2), their confidence in executing counseling tasks, particularly personal adjustment skills, was moderate (Table 3). This importance–confidence gap mirrors international findings in which audiologists conceptually endorse counseling but report feeling less prepared to address the psychosocial dimensions of hearing loss. Studies have long documented a tendency for audiologists to emphasize informational counseling while engaging less extensively in psychosocial discussions during appointments (7, 8), a trend similarly reflected here in the lower confidence for discussing patient and family emotions. In line with this pattern, participants assigned comparatively lower importance to explaining the psychosocial sequelae of hearing loss, suggesting that broader discussions of long-term psychosocial impact may receive less emphasis within routine clinical counseling.

Building on these overall patterns, demographic analyses reinforced the developmental nature of counseling competence. Audiologists in the older age group and those with more than 10 years of experience demonstrated greater confidence (Table 7), supporting literature indicating that counseling comfort increases with accumulated clinical exposure (9, 17). Nonetheless, the absence of significant differences in perception across demographic groups (Table 6) suggests that the value placed on counseling is universally recognized, independent of age, gender, education, or work setting. This is consistent with international trends where audiologists consistently express strong beliefs about the importance of counseling, even when their practical skills vary (9, 11, 13, 14). At the skill-specific level, participants reported only moderate confidence in assessing psychosocial challenges and determining the need for psychological referral, indicating uncertainty in identifying when additional support may be required.

Within this broader context, training emerged as the most consistently associated factor across study outcomes. Participants reported varied preparation, with only a small proportion having completed a dedicated counseling course and the majority receiving either limited or no training during their academic studies or careers (Table 5). This aligns with previous reviews documenting inconsistent counseling curricula across audiology programs and limited depth of instruction in psychosocial methods (16, 24). Importantly, the regression analyses demonstrated that training variables were the strongest independent associations with both confidence and perceived barriers. A higher self-identified need for further training was associated with lower confidence, whereas participation in workshops was associated with higher confidence (Table 9). Similarly, greater perceived adequacy of training was independently associated with fewer perceived barriers, and the absence of training was associated with higher barriers (Table 11). Although barriers were generally rated as low, these associations suggest that the quality and presence of training may influence how clinicians experience counseling-related challenges, particularly in early career stages.

Participants also reported greater comfort in assessing patients' understanding of technical information related to hearing aids and cochlear implants, further reinforcing the tendency for audiologists to feel more secure in informational counseling than in psychosocial and personal adjustment skills. These findings are reinforced by evidence suggesting that structured, experiential learning methods, such as simulations, role-plays, and supervised practice, may provide important practice opportunities to enhance counseling readiness in audiology (24, 25). It should also be noted that the psychosocial and personal adjustment aspects of counseling were assessed using a limited number of survey items, which may not fully capture the complexity of counseling practices in clinical audiology. This measurement constraint should be considered when interpreting the confidence levels reported in these domains.

The observed association between confidence and perceived importance (Table 10) should be interpreted with caution. Because both constructs were measured using self-reported ratings within the same survey at a single time point, the association may partly reflect shared method variance rather than a substantively meaningful relationship. Accordingly, the finding does not indicate a directional or bidirectional effect between confidence and perception, but rather a concurrent association influenced by the measurement approach. Confidence was the only variable associated with perception scores in the multivariable model; however, this relationship should be considered exploratory and interpreted within the methodological constraints of the study. Similar patterns have been reported internationally, where increased professional competence is linked to greater engagement in psychosocially informed care (11). However, given the cross-sectional design, these findings reflect associations rather than directional effects.

Placing these findings within the national educational context, variability in the depth and structure of counseling training during undergraduate education may contribute to the observed patterns in confidence and perceived barriers. Publicly available program descriptions suggest that structured counseling instruction is not uniformly emphasized across institutions. Given that the Bachelor of Science degree is the entry-to-practice qualification in Saudi Arabia, differences in exposure to structured counseling education may influence graduates' preparedness to provide both informational and personal adjustment counseling. While this study did not directly evaluate specific academic curricula, the consistent associations between perceived training adequacy and study outcomes underscore the importance of competency-based counseling education.

International literature reflects similar concerns, noting that when counseling education is limited or inconsistently taught, clinicians report lower confidence and reduced engagement with personal adjustment aspects of hearing loss management (9, 10, 16). Observational work has further demonstrated that audiologists frequently miss opportunities to respond to patients' psychosocial concerns (7, 8), a pattern that may be reinforced when formal training in counseling skills is limited. The identification of patient resistance and limited family involvement as moderately rated barriers may reflect challenges related to readiness for change, denial, or limited understanding of the counseling process, particularly in psychosocially sensitive stages of hearing loss management. It is important to clarify that personal adjustment counseling within audiology involves supportive communication, identification of psychosocial concerns, and appropriate referral when needed, rather than providing psychotherapy-level interventions. Complex psychological conditions remain within the scope of trained mental health professionals.

A methodological consideration relevant to the interpretation of these findings concerns the internal consistency of the perception scale used in this study. The perception domain demonstrated borderline internal consistency (Cronbach's α = 0.64), suggesting some heterogeneity across the items included within this construct. This may reflect that the perception items capture several related aspects of clinicians' views on counseling, such as its perceived importance, scope, and role within audiologic care, rather than a single tightly unified dimension. Accordingly, the perception findings should be interpreted with appropriate caution. Future research may benefit from refining or expanding the items within this domain to improve measurement consistency and to further clarify how audiologists conceptualize counseling within clinical practice.

Collectively, these findings highlight a meaningful opportunity to strengthen counseling preparation within audiology education and professional development. Greater consistency in structured counseling coursework may help ensure more uniform preparation of graduates. This aligns with long-standing recommendations to strengthen counseling training through structured curricula and skill-based instruction, given that variability in preparation may contribute to differences in clinical practice (16, 17). Incorporating experiential learning methods, such as simulation and supervised clinical counseling experiences, may further enhance competence and confidence (24, 25). Because BSc-level clinicians make up the majority of the national audiology workforce, strengthening undergraduate counseling preparation may support improved patient care, reduce perceived professional barriers, and promote more holistic and psychosocially responsive audiologic practice.

Overall, these findings position audiologists in Saudi Arabia within a broader international pattern characterized by strong conceptual endorsement of counseling, moderate confidence in practice, and an identified need for structured, competency-based training. Strengthening counseling preparation at both academic and clinical levels may contribute to enhancing patient-centered care and supporting audiologists in delivering comprehensive services consistent with professional guidelines.

## 5 Limitations, recommendations and future directions

This study has several limitations that should be considered when interpreting the findings. The sample, although diverse in age and work settings, was predominantly composed of younger clinicians and highly concentrated in the central region, which may limit generalizability to the broader national audiology workforce. In particular, the majority of respondents were early- to mid-career audiologists, and senior clinicians were underrepresented. Therefore, the findings may more strongly reflect the experiences and perspectives of early-career practitioners. The cross-sectional design also prevents causal inferences about the relationships between training, confidence, and perceived barriers. Additionally, self-reported data may be subject to response bias, including potential social desirability effects, particularly in areas involving self-evaluation of competence or perceived professional responsibilities. Moreover, as perception and confidence were measured using self-reported scales within the same survey at a single time point, their observed associations may be influenced by shared method variance rather than reflecting substantive relationships. Furthermore, psychosocial and personal adjustment aspects of counseling were assessed using a limited number of survey items, which may not fully capture the depth and complexity of counseling practices within clinical audiology. In addition, the perception scale demonstrated borderline internal consistency (Cronbach's α = 0.64), which may reflect variability across items within this domain. These measurement constraints should be considered when interpreting the findings, as they may limit the precision with which clinicians' perceptions of counseling are captured.

Although multiple regression analyses were conducted to examine independent associations among study variables, the sample size relative to the number of predictors warrants cautious interpretation. The regression findings should therefore be considered exploratory, and larger studies are needed to confirm these relationships.

Beyond undergraduate education, the findings also highlight the need to support practicing audiologists through targeted continuing professional development. Workshops and short courses focusing on personal adjustment counseling, psychosocial screening, referral decision-making, family-centered counseling, and strategies for managing patient resistance may help strengthen confidence and mitigate specific counseling-related challenges among early- and mid-career clinicians.

Future research should consider mixed-methods or longitudinal designs to capture changes in counseling skills and perceptions over time, especially following structured training interventions. Qualitative studies may also provide richer insight into how audiologists navigate counseling interactions in real practice. Importantly, the findings of this study suggest variability in the extent to which structured counseling education is formally incorporated within undergraduate audiology programs in Saudi Arabia. Therefore, future efforts should focus on strengthening and standardizing counseling education, including the development of dedicated courses or expanded curricular content and the integration of experiential training methods such as simulation, role-play, and supervised counseling practice. Enhancing undergraduate preparation in this area is especially important given that the Bachelor's degree is the practicing qualification in the country.

## 6 Conclusions

In summary, audiologists in Saudi Arabia strongly value counseling but report only moderate confidence in delivering it, particularly in personal adjustment and emotionally focused interactions. Training was independently associated with both confidence and perceived barriers, underscoring the importance of structured and comprehensive counseling education. Strengthening training opportunities and enhancing undergraduate preparation may support more holistic, patient-centered audiologic care and better address the psychosocial needs of individuals with hearing loss.

## Data Availability

The raw data supporting the conclusions of this article will be made available by the authors on reasonable request, without undue reservation.
