# Supplementary material for: Counseling in audiology practice in Saudi Arabia: perceptions, confidence, training experiences, and barriers – a cross-sectional survey
Source: Front Med (Lausanne). 2026 Apr 21;13:1792266. doi: 10.3389/fmed.2026.1792266 (PMC13138957; doi:10.3389/fmed.2026.1792266)
Supplement: Supplementary file 1 [file Table_1.docx]

**Supplementary material: Survey**

**Section 1: Demographic Information**

- **Age**
- 24-34
- 35-45
- 46- 55
- 56 and older
- **Gender**
  - Male
  - Female
- **Region of residency**
- Central
- Western
- Eastern
- Southern
- Northern
- **Level of education**
  - Bachelor's Degree
  - Master's Degree
  - Doctoral Degree
- **Years of clinical experience**
  - Less than 1 year
  - 1-5 years
  - 6-10 years
  - More than 10 years
- **Work setting**
  - Private Hospital/clinic
  - Public Hospital
  - Both

**Section 2: perceptions of counseling aspects**

How important do you believe the following counseling aspects are for effective patient care in audiology?

| Item | Not important (1) | Slightly important (2) | Moderately important  (3) | Extremely important  (4) |
| --- | --- | --- | --- | --- |
| Explaining the diagnosis of hearing loss and related symptoms to the patient |  |  |  |  |
| Explaining the expectations (e.g., outcomes, process, realistic goals) to the patient |  |  |  |  |
| Explaining the technical information (related to HAs\CIs) to the patients |  |  |  |  |
| Discussing patients’ and their families’ emotions when receiving a diagnosis (e.g., sadness, anger, and confusion) |  |  |  |  |
| Explaining the common sociological\psychological sequelae of hearing loss (e.g., social isolation, depression, stress) to the patient |  |  |  |  |
| Discussing psychological referral for those who experience significant mental health challenges |  |  |  |  |

**Section 3: Training received in Counseling**

1. **What types of training specific to the knowledge and skills for counseling did you receive during your undergraduate or postgraduate education?**

- No training received
- Included in 1–2 audiology content courses (e.g., hearing aids)
- Included in several audiology content courses
- One or more courses specific to counseling in audiology

1. **How often did you receive training specific to counseling during your clinical career?**

- No training received
- Rarely (e.g., once or twice during your career)
- Occasionally (e.g., a few times throughout your career)
- Frequently (e.g., at least once a year)
- Very Frequently (e.g., more than once a year)

1. **What types of counseling training have you received through your professional development? (Select all that apply)**

- None
- Workshops
- Online courses
- Peer mentorship or shadowing
- Other (please specify)

1. **How would you rate the adequacy of your training in counseling?**

- Very Inadequate
- Inadequate
- Neutral
- Adequate
- Very Adequate

1. **Do you feel the need for additional training in audiological counseling?**

- No, I feel fully competent
- No, but occasional refreshers would be helpful
- Yes, I would benefit from some additional training
- Yes, I need extensive training to feel confident

**Section 4: Confidence in counseling skills**

Rate your confidence in your ability to perform each of the following skills.

| Item | Not Confident  (1) | Somewhat Confident  (2) | Confident  (3) | Very Confident  (4) |
| --- | --- | --- | --- | --- |
| Talking about the patients\ patients' family' emotions (e.g., sadness, anger) |  |  |  |  |
| Assessing the patient's understanding of technical information (HAs\CIs) |  |  |  |  |
| Assessing the presence of psychosocial challenges |  |  |  |  |
| Explaining the diagnosis of hearing loss and related symptoms to the patients |  |  |  |  |
| Explaining the expectations (e.g., outcomes, process, realistic goals) to the patient |  |  |  |  |
| Assessing the need for psychological referral |  |  |  |  |

**Section 5: Barriers to Effective Counseling**

Indicate how challenging you find each item when counseling patients and their families.

| Item | Not challenging  (1) | Somewhat challenging  (2) | Challenging  (3) | Very challenging  (4) |
| --- | --- | --- | --- | --- |
| Having sufficient time to address the patient's emotional needs |  |  |  |  |
| Having the confidence to perform proper counseling |  |  |  |  |
| Not receiving enough counseling training |  |  |  |  |
| Limited educational resources for the patients |  |  |  |  |
| The patient's resistance to counseling (e.g., Denial, or low motivation) |  |  |  |  |
| Managing patient and family expectations |  |  |  |  |
| Limited family involvement (e.g., Lack of family\partner support) |  |  |  |  |
